# Effects of normal aging on the mouse retina assessed by full-field flash and flicker electroretinography

**DOI:** 10.1038/s41598-023-35996-7

**Published:** 2023-05-31

**Authors:** Jason C. Park, Oksana Persidina, Giri Balasubramanian, Tara Nguyen, Anubhav Pradeep, John R. Hetling, J. Jason McAnany

**Affiliations:** 1 Department of Ophthalmology and Visual Sciences, University of Illinois at Chicago, 1855 W. Taylor St., MC/648, Chicago, IL 60612 USA; 2 Department of Bioengineering, University of Illinois at Chicago, 851 South Morgan St., Chicago, IL 60607 USA

**Keywords:** Preclinical research, Neuroscience, Physiology

## Abstract

Changes in the full-field flash and flicker electroretinogram (ERG) that accompany normal aging were evaluated in mice. ERGs were recorded from a single cohort of C57BL/6J mice from 5 to 70 weeks of age using conventional techniques. Dark-adapted ERGs were recorded for flash luminances of − 3.0 to 1.5 log cd-s-m^−2^; a- and b-wave amplitude and implicit time (IT) were calculated from these responses. In addition, light-adapted flicker ERGs elicited by sinusoidally modulated light were measured for temporal frequencies of 2 to 31 Hz. Amplitudes and phases were extracted from the flicker responses using Fourier analysis. Linear quantile mixed models were used for statistical comparisons of the effects of age on amplitude and timing. There was a significant decrease in a-wave amplitude (*p* < 0.001) and b-wave amplitude (*p* < 0.001) over the 65 week study. From 5 to 70 weeks, the a- and b-wave amplitudes decreased by a factor of approximately 2. There was a small (2–14 ms), but significant (*p* < 0.001), delay in a- and b-wave IT over the 65 week study. There was also a significant decrease in fundamental amplitude (factor of 1.8, *p* < 0.001) and second harmonic amplitude (factor of 1.5, *p* < 0.001) over time. There were no significant age-related effects on the phase of these components (both *p* > 0.06). These results indicate that age scales the single flash and flicker ERG similarly, reducing response amplitude by a factor of approximately 2, from 5 to 70 weeks, with small or no effect on response timing. These data may be useful for guiding future longitudinal pre-clinical therapeutic studies.

## Introduction

The electroretinogram (ERG) is the mass electrical response of the retina in response to light stimuli. This technique has become an essential tool for evaluating retinal function in pre-clinical models of retinal disease and ocular therapeutics. ERGs elicited by brief, full-field flashes of light are commonly recorded under dark-adapted conditions to study rod-pathway function and under light-adapted conditions to study cone-pathway function. As discussed in detail elsewhere^1^, the a-wave of the flash ERG is predominately a measure of photoreceptor function, whereas the b-wave generally reflects post-receptor function. The ERG elicited by flickering light has been used as an approach to study cone-pathway function, although flicker stimuli are less commonly used in murine models. Previous work in mice has indicated that the cellular source of the flicker ERG may depend on stimulus temporal frequency, with moderate temporal frequencies (5–15 Hz) being ON-pathway dominated and high temporal frequencies (above 15 Hz) being OFF-pathway dominated^2^.

Age is an essential biological variable that affects the ERG recorded from both humans^3,4^ and mice^5–8^. Despite the importance of age, relatively few studies have examined the effect of age on the mouse ERG. Understanding the natural history of ERG changes is important for the design of longitudinal therapeutic studies. Of the available reports, Williams and Jacobs^6^ observed smaller 12.5 Hz flicker ERG amplitudes for C57BL/6 mice that were 30 months of age compared to mice that were 1 month of age. Cone photoreceptor death did not appear to account directly for the photopic flicker ERG differences, as they found no difference in cone density or in opsin gene transcription over this timespan. Gresh et al.^7^ examined flicker ERGs across a broader range of flicker frequencies, 1 to 30 Hz, in C57BL/6 and BALB/c mice and found smaller flicker ERG amplitudes at months 4 and 17 compared to month 1, with minimal differences in cone number among the three age groups. They did not report the effect of age on flicker ERG amplitude or timing for individual stimulus frequencies, so it remains unclear how age affects moderate temporal frequency responses (possibly ON-pathway-dominated) versus high temporal frequency responses (possibly OFF-pathway-dominated).

Consistent with their flicker ERG data, Gresh et al.^7^ found smaller dark-adapted single flash ERG amplitudes at months 4 and 17 compared to month 1, and that rod photoreceptor number and rhodopsin content were reduced in the older group. A study of the pigmented hybrid B6D2F1/J mouse strain, which has a lifespan that is typically longer than the C57BL/6 strain, also found reduced scotopic flash ERGs in 2.5 year-old mice compared to 4-month old mice^5^. In the 2.5 year old mice, a reduced number of rod photoreceptors, receptor outer segment shortening, and decreased opsin content were observed, compared to the 4-month group^5^. Consistent with these reports, Li et al.^8^ observed smaller photopic and scotopic single flash ERG amplitudes for 12 month-old C57BL/6 mice compared to 2-month old mice. However, Li et al.^8^ reported no significant difference in photoreceptor density over this timespan. Given the similar declines in rod and cone photoreceptor function in the absence of apparent cell loss, it was proposed that a decrease in ocular impedance with age could explain the ERG amplitude attenuation^8^. That is, the ERG is a field potential and a change in the electrical properties of the tissues that conduct the response can impact the voltage recorded at the cornea. Taken together, previous studies are generally in agreement that rod- and cone-pathway ERG amplitudes decrease with age, but the extent to which the ERG changes are related to photoreceptor cell death is less clear.

The goal of the present study was to gain a more complete understanding of the natural history of ERG changes due to normal aging in the C57BL/6 mouse, a commonly used strain in pre-clinical Ophthalmology research. As opposed to the studies reviewed above, ERGs were recorded repeatedly on a fine timescale from 5 to 70 weeks of age in the same cohort of mice. Following the same mice longitudinally in a repeated measures design helps to minimize inter-mouse differences as a source of variability. Dark-adapted flash ERGs were recorded across a broad range of luminance and light-adapted flicker ERGs were recorded across a broad range of stimulus frequency. Changes in the fundamental (linear response component) and second harmonic (nonlinear response component) of the flicker ERG were assessed over time, as the effect of age on these components has not been reported. Finally, the ocular impedance of mice at 8 and 70 weeks of age was quantified to test the hypothesis that a decrease in ocular impedance underlies the age-related ERG amplitude loss.

## Methods

### Animals

This study was approved by the University of Illinois at Chicago Animal Care Committee and was conducted in accordance with the ARRIVE guidelines. All methods were performed in accordance with institutional and national guidelines and regulations. Ten male C57BL/6J mice were obtained from the Jackson Laboratory (Strain# 000664) at 4 weeks of age. Mice were housed 5 to a cage under conventional lighting conditions (14/10 h light–dark cycle) and fed a standard laboratory diet. ERGs were measured at 5 weeks of age, then every 5 weeks until the age of 30 weeks. From the age of 30 to 70 weeks, ERGs were obtained every 10 weeks. Not all mice survived the 65 week study: N = 10 from weeks 5 to 15; N = 9 at week 20; N = 7 at weeks 50 and 60; N = 6 at week 70.

### ERG apparatus, stimuli, procedure, and analysis

Stimuli were generated by and presented with a Celeris rodent electrophysiology system (Diagnosys LLC, Lowell, MA). The flash stimuli consisted of brief (≤ 4 ms), achromatic, full-field luminance that ranged from − 3.0 to 1.5 log cd-s-m^−2^. The flicker stimuli consisted of sinusoidal modulation of a uniform field. The mean luminance of the flicker was 200 cd/m^2^, consisting of 100 cd/m^2^ each of 632- and 516-nm stimuli. The stimulus luminance was maintained at this mean level between presentations of the flicker. The luminance levels used in previous rodent flicker ERG studies vary considerably^9,10^ (e.g. from 60 to 350 cd/m^2^). Our selection of 210 cd/m^2^ has been used to suppress the rod response in human flicker ERG studies^11^ and helps ensure that ERG responses reflect the activity of cone-mediated pathways^12^. Full-field flicker was presented against a steady, rod-saturating adapting field of 10 cd/m^2^, generated by LEDs with a peak wavelength of 453 nm. The stimulus contrast was 100% (Michelson) and the flicker frequencies ranged from 2 to 31 Hz in 9 steps spaced approximately 0.15 log units apart.

Mice were dark-adapted for 2-h prior to testing. Under dim red illumination, anesthesia was administered by intraperitoneal injection of ketamine (100 mg/kg) and xylazine (5 mg/kg). Pupils were fully dilated with tropicamide (0.5%) eye drops. ERGs were recorded using the combined stimulator/electrode probe of the Celeris system (Diagnosys, LLC) that was placed in contact with each cornea and aligned with the center of the pupils. Under this arrangement, stimuli were delivered to one eye at a time, with the fellow, unstimulated, eye serving as the reference. Data obtained from the left and right eyes were averaged for analysis. Amplifier bandpass settings were 0.125 to 300 Hz and the sampling frequency was 2 kHz.

Three single flash responses for each stimulus luminance were obtained and averaged for analysis. Following the dark-adapted measurements, the mice were light-adapted for two-minutes to the 210 cd/m^2^ adapting field described above (sum of 100 cd/m^2^ red, 100 cd/m^2^ green, and 10 cd/m^2^ blue). Following light-adaptation, 3 responses were obtained and averaged for analysis for each stimulus flicker frequency. Waveforms were exported for analysis in MATLAB. For the flash responses, the a- and b-wave amplitudes and implicit times (ITs) were calculated according to convention at the peaks and troughs^13^. Of note, a similar pattern of data was obtained for a-wave measurements made at a fixed time point, rather than at the trough. The amplitude and phase of the ERG fundamental component and second harmonic were extracted by Fourier analysis for each stimulus frequency. Only flicker responses with a signal to noise ratio of 2.82 were included in the analyses, according to convention and discussed elsewhere^14^.

### Ocular impedance measurements

Impedance of the corneal–buccal current pathway was evaluated in 5 mice following the completion of the 70-week study and in 5 mice that were 8 weeks of age; the 8-week group was not involved in the longitudinal study. The age of 8-weeks was selected to approximate the size and weight of the 70 week group, as mice grow rapidly before approximately 6 weeks of age. The approach to measure ocular impedance was similar to that described elsewhere.^15^ In brief, the corneal–buccal impedance was measured with a voltage divider using two electrodes: (1) a Ag/AgCl pellet electrode placed in contact with the cornea using Refresh Celluvisc tears (Allergan, Inc) as an electrolyte; (2) a Ag/AgCl pellet electrode placed in the mouth. Corneal–buccal impedance was defined as the combined impedance of the current pathway and the electrodes. The two-electrode setup was placed in series with a known resistor (47 kΩ). An input (a 10 Hz, 100 mV sinusoidal wave) was supplied using a function generator. The input frequency of 10 Hz is near the middle of the frequencies used for the flicker ERG measurements and is within the frequency band of the single flash responses. Corneal–buccal impedance was calculated by measuring the voltage output at the known resistance.

### Statistical analyses

Statistical analyses were conducted using R (version 4.0.2; R Core Team, Vienna, Austria) and SigmaPlot (version 12; San Jose, CA, USA). The amplitude and IT distributions were evaluated for normality using Shapiro–Wilk tests; the amplitude and IT distributions were found to be skewed significantly. Consequently, linear quantile mixed models (LQMMs)^16^, which are based on median values, were used to evaluate the effect of age on amplitude and implicit time. In each model, age was included as the main independent variable while adjusting for flash luminance or flicker frequency. A random intercept was added at the animal level to account for the repeated measures obtained from each animal. For waveform components that returned a significant difference (*p* < 0.05) due to age, univariate linear quantile models (LQMs)^17,18^ were developed to determine whether the response component at a particular age differed from the response at the first measurement (week 5). These analyses were stratified by response component (a-wave, b-wave, flicker fundamental, flicker second harmonic). The impedance data were found to be normally distributed (Shapiro–Wilk; *p* = 0.81). Consequently, a t-test was used to compare impedance values for the 8 and 70 week-old mice.

## Results

### Dark-adapted flash ERG

**Figure 1:**
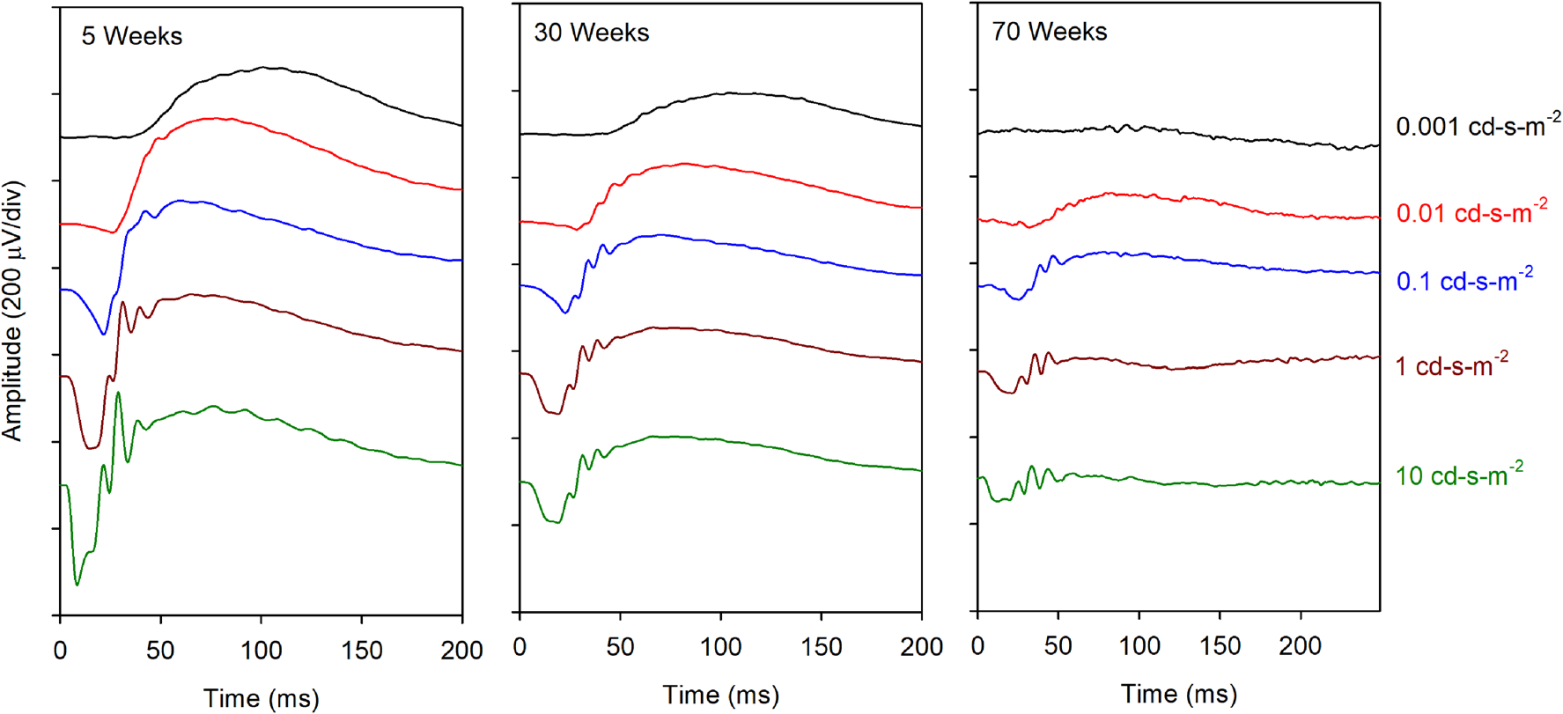
Mean dark-adapted flash waveforms elicited by different flash luminance (indicated to the right) for mice that were 5 weeks (left) 30 weeks (middle) and 70 weeks (right) of age.

**Figure 2:**
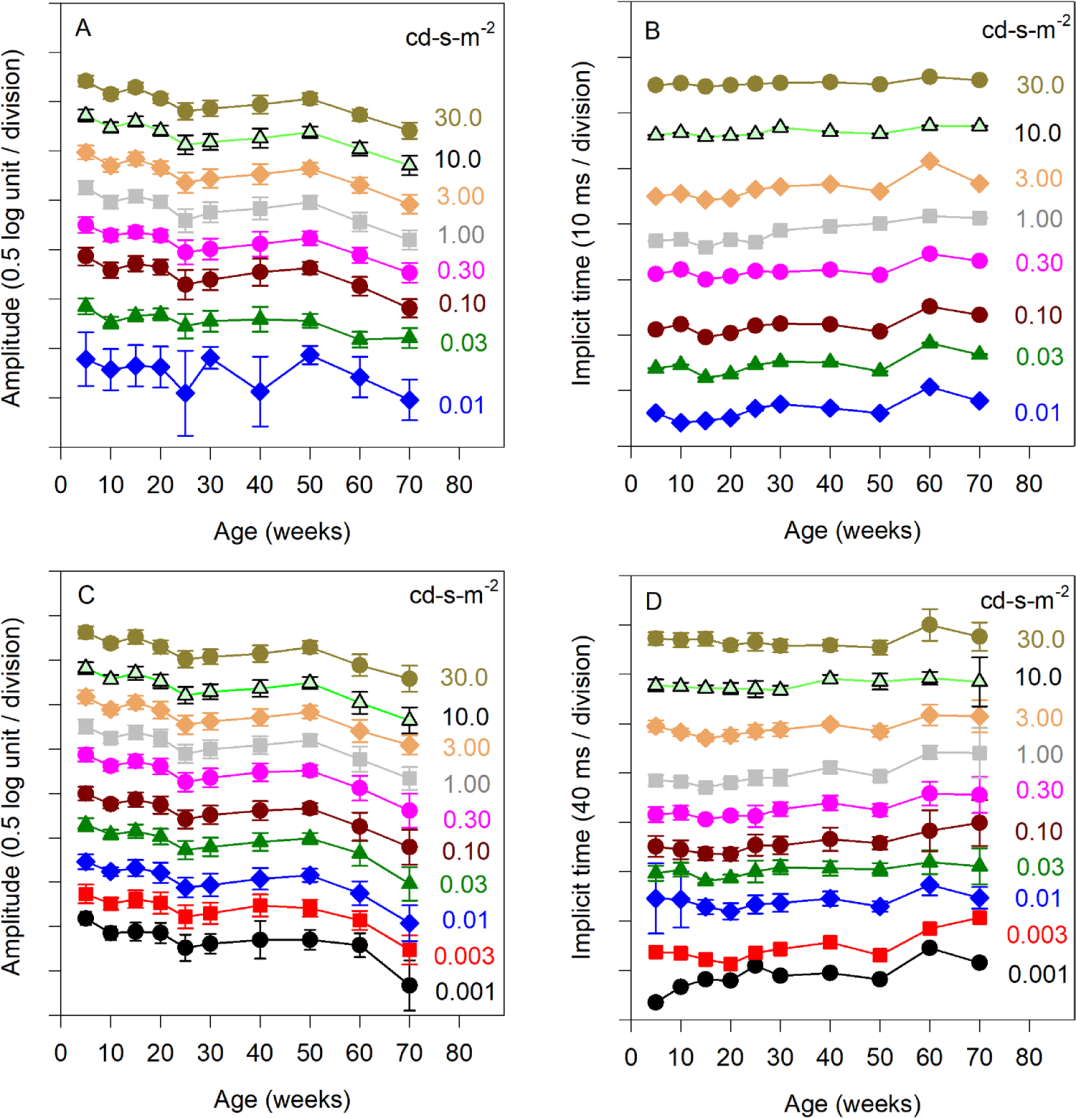
Mean (± SEM) log a-wave amplitude (**A**), a-wave IT (**B**), log b-wave amplitude (**C**), and b-wave IT (**D**) are plotted as a function of age. Each function represents a different flash luminance (indicated to the right). Each function is displaced vertically to allow inspection of the individual flash luminance data. Note that a-waves were not apparent for low luminance flashes and these data are therefore not shown in panels A and B.

Figure 1 shows the mean dark-adapted single flash responses elicited by a subset of the flash luminances tested (0.001, 0.01, 0.1, 1.0, and 10.0 cd-s-m^2^) recorded at 5 weeks (earliest age), 30 weeks (middle), and 70 weeks (final measurement) of age. It is clear from these average waveforms that the ERG became smaller with increasing age. For the lowest luminance flash (black), the mean response was essentially extinguished at 70 weeks of age. For the highest luminance flash, the a-wave was markedly attenuated and the b-wave was nearly absent. Indeed, the decrease in amplitude with increasing age was clear for each of the five stimulus luminances shown in Fig. 1. This figure is intended to illustrate the general waveform shape of the single flash responses and how they change with age; the a- and b-wave amplitudes and implicit times were extracted from the waveforms are quantified in Fig. 2.

Figure 2 shows the mean (± SEM) a-wave amplitude (A), a-wave IT (B), b-wave amplitude (C), and b-wave IT (D) as a function of age. Each function represents a different flash luminance, as indicated to the right of each plot. Data for each flash luminance are displaced vertically to permit visualization. Amplitudes of the a- and b-waves decreased with age from 5 to 30 weeks, were generally constant from weeks 30 to 50 (perhaps slightly increasing), then decreased from weeks 50 to 70. This pattern was observed for each stimulus flash strength for both the a- and b-wave amplitudes. Figure 2B,D show subtle effects of age on a- and b-wave IT that were somewhat more apparent for low luminance flashes. The effect of age on amplitude and IT was evaluated statistically using LQMMs, as described in the Methods. LQMMs indicated that there was a significant effect of age on ERG amplitude (median a-wave decrease of 0.22 log units [*p* < 0.001] and b-wave decrease of 0.37 log units [*p* < 0.001] over the 65 week study). LQMMs indicated that there was a significant effect of age on IT (median a-wave delay of 2.4 ms [*p* < 0.001] and b-wave delay of 13.7 ms [*p* < 0.001] over the 65 week study). LQMs indicated amplitude loss and timing delays for the a- and b-waves for several individual time-points, compared to the initial measurement at week 5. These analyses are presented in Table 1 for comparisons that were statistically significant (all other *p* > 0.05).

**Table 1:** Significant findings of the LQM model.

Age comparison; week 5 versus week:	Median difference estimate (log µV loss)	*p*-value
a-wave amplitude		
25	0.25	0.001
30	0.19	0.006
60	0.27	< 0.0001
70	0.41	< 0.0001
b-wave amplitude		
10	0.14	0.010
20	0.12	0.046
25	0.32	< 0.0001
30	0.27	0.002
40	0.21	0.021
50	0.19	0.009
60	0.34	< 0.0001
70	0.61	< 0.0001

Age comparison week 5 verus week	Median difference estimate (ms delay)	*p*-value
a-wave IT delay		
10	0.86	0.017
60	3.18	< 0.0001
b-wave IT delay		
20	4.53	0.040
60	15.33	< 0.0001

Age comparison week 5 versus week	Median difference estimate (log µV loss)	*p*-value
Fundamental amplitude		
15	0.12	0.005
20	0.14	0.001
25	0.20	< 0.0001
30	0.26	< 0.0001
40	0.28	< 0.0001
50	0.14	0.003
60	0.29	< 0.0001
0	0.37	< 0.0001
Second harmonic amplitude		
10	0.10	0.015
25	0.27	< 0.0001
40	0.19	0.021
50	0.16	0.009
60	0.17	< 0.0001
70	0.31	< 0.0001

An alternative approach to analyze the effect of age on the b-wave is presented in the Supplemental Materials (Fig. S1). In brief, b-wave amplitude was plotted as a function of log flash luminance and fit with Naka-Rushton functions to derive *V*_*max*_ (maximum b-wave amplitude) and *K* (semi-saturation constant). Consistent with Fig. 2, this analysis showed that *V*_*max*_ decreased with age over approximately 5–30 weeks, slightly increased from weeks 30–50, then decreased sharply after 50 weeks of age. In contrast, b-wave sensitivity (log *K*) was generally independent of age. These results are consistent with those obtained from human subjects^3^, which also showed considerable loss of *V*_*max*_ from age 20 to 68, with minimal increase in *K*.

### Flicker responses

**Figure 3:**
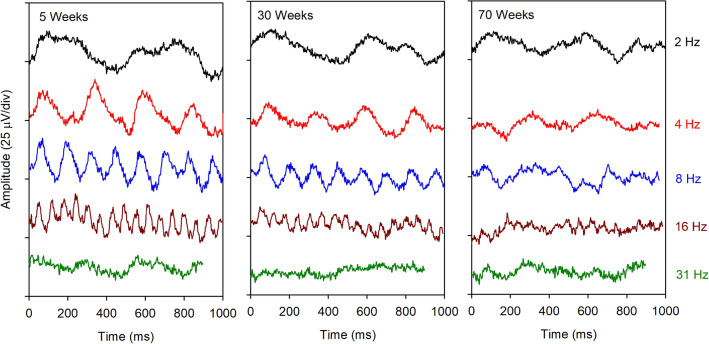
Mean light-adapted flicker waveforms elicited by different flicker frequencies (indicated to the right) for mice that were 5 weeks (left) 30 weeks (middle) and 70 weeks (right) of age.

**Figure 4:**
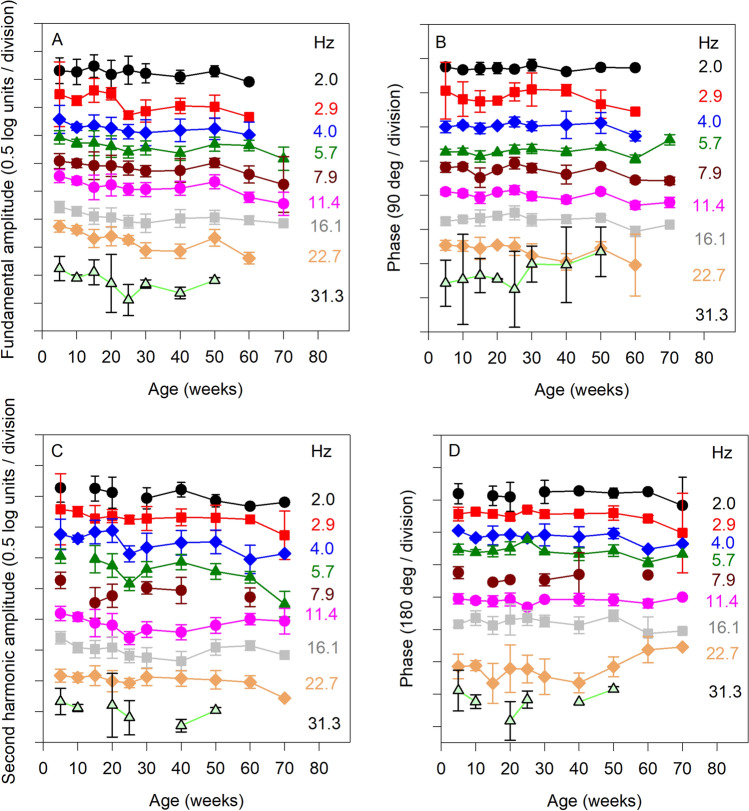
Mean (± SEM) log fundamental flicker amplitude (**A**), fundamental phase (**B**), log second harmonic amplitude (**C**), and second harmonic phase (**D**) are plotted as a function of age. Missing data points indicate stimulus frequencies for which fewer than three mice had a response with a SNR > 2.82. Other conventions are as in Fig. 2.

Figure 3 shows the mean light-adapted flicker responses elicited by 2, 4, 8, 16, and 31 Hz stimuli recorded at 5 weeks (earliest age), 30 weeks (middle), and 70 weeks (final measurement) of age. As for the single flash responses, it is clear from these average flicker waveforms that the ERG became smaller with increasing age. The response attenuation was most apparent for the 4 to 31 Hz stimuli, where periodic responses at 70 weeks were difficult to discern. The response at 2 Hz appeared to be less age-dependent, but the response at this flicker frequency was contaminated by breathing artifacts, which made the stimulus-driven response difficult to discern from the waveform. This figure is intended to illustrate the general waveform shapes of the flicker responses across frequency; the amplitudes and phases are quantified in Fig. 4.

Figure 4 shows the mean (± SEM) fundamental amplitude (A), fundamental phase (B), second harmonic amplitude (C) and second harmonic phase (D) as a function of age. Each function represents a different flicker frequency, as indicated to the right of each plot. Data for each frequency are displaced vertically to permit visualization. The pattern of flicker ERG changes over time were highly similar to the pattern of single flash ERGs over time. Specifically, the amplitude of the flicker ERG tended to decrease with age from 5 to 30 weeks, was generally constant from weeks 30 to 50 (perhaps slightly increasing), then decreased from weeks 50 to 70. This pattern was observed for each flicker frequency for both the fundamental and second harmonic amplitudes. The LQMMs indicated that there was a significant effect of age on the flicker ERG amplitude (median fundamental decrease of 0.27 log units [*p* < 0.001] and second harmonic decrease of 0.17 log units [*p* < 0.001] over the 65 week study). LQMs indicated amplitude loss for the fundamental and second harmonic for several individual time-points, compared to the initial measurement at week 5. These analyses are presented in Table 1. Figure 4B,D show little to no effect of age on the phase of the flicker responses. LQMMs indicated no significant effect of age on the fundamental phase (median delay of 12.1 deg over the 65 week study; *p* = 0.064) or second harmonic phase (median delay of 6.1 deg over the 65 week study; *p* = 0.62).

### Flash versus flicker responses over time

**Figure 5:**
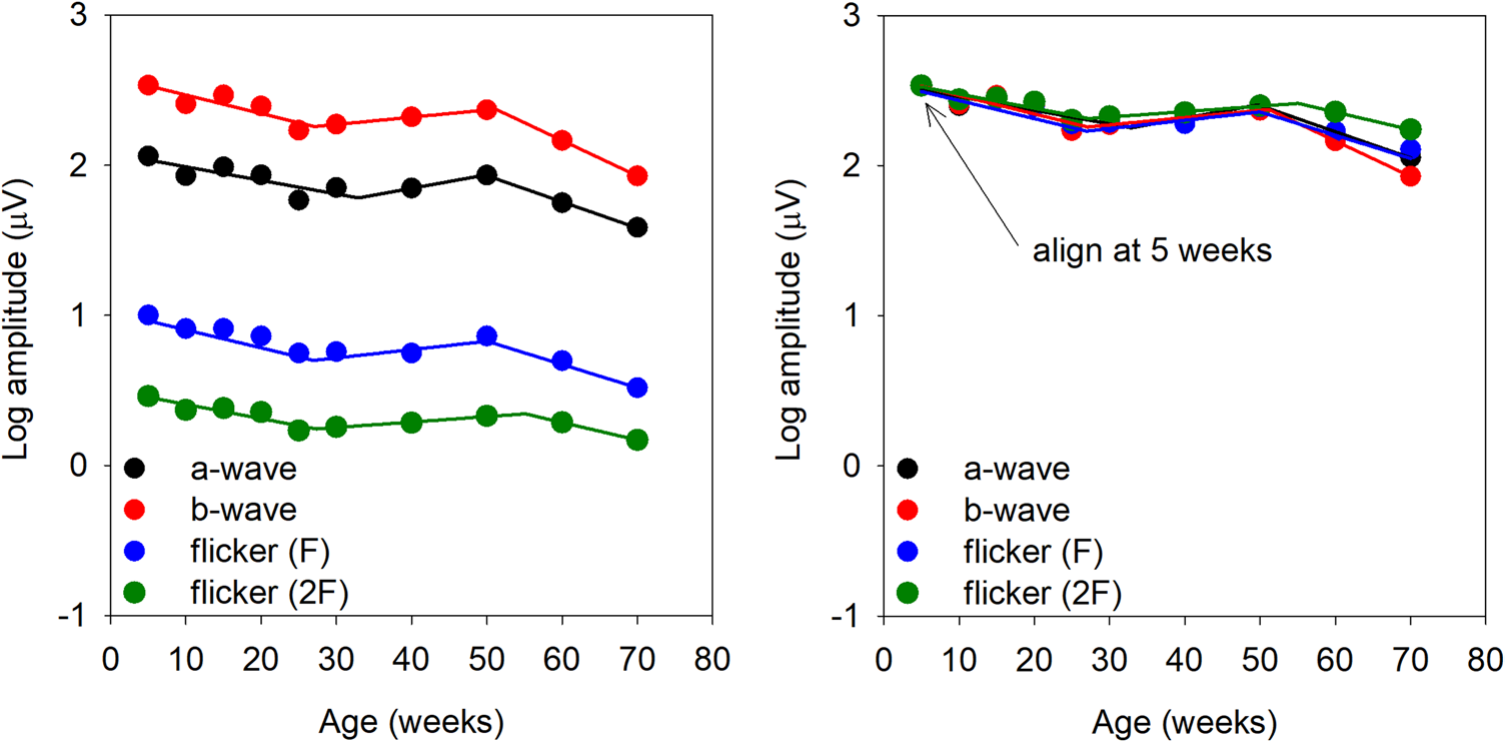
Mean log amplitude for the a-wave, b-wave, fundamental flicker response, and second harmonic of the flicker response are plotted as a function of age (panel A). The data are fit piecewise with three linear segments. Panel B shows data replotted from panel A after shifting the functions vertically to align at the 5-week time point.

Figure 5 (left) shows the a- and b-wave amplitudes (averaged over all flash luminances) and the fundamental and second harmonic flicker amplitudes (averaged over all stimulus frequencies). The data are fit with piecewise with a function that had three linear segments. For each stimulus type, the initial linear function showed a decrease in amplitude with age over approximately 5–30 weeks, a slight increase from weeks 30–50, and a decrease after 50 weeks of age. The right panel replots the amplitude data from the left panel, with alignment at week 5. This panel highlights the finding that the pattern of amplitude change over time is nearly identical for the a-wave, b-wave, fundamental flicker, and second harmonic flicker responses.

### Ocular impedance

**Figure 6:**
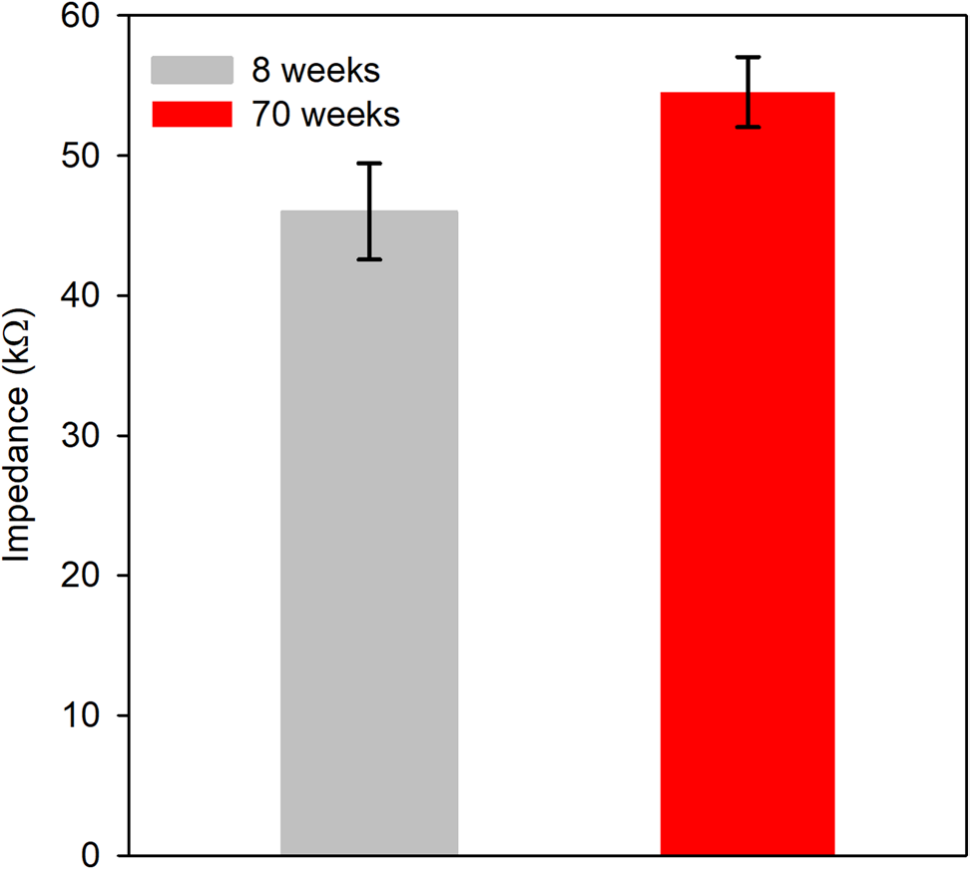
Mean (± SEM) ocular impedance for mice that were 8 weeks old (gray) and 70 weeks old (red).

Figure 6 shows the mean (± SEM) ocular impedance measured at 8 weeks (gray) and 70 weeks (red) of age. Mean impedance was somewhat greater in the older mice compared to the younger mice, but a t-test indicated no statistically significant difference between groups (t = 2.0, *p* = 0.08). These findings do not support the hypothesis that reduced impedance in aged mice underlies the age-related ERG amplitude reduction, as discussed further below.

## Discussion

The purpose of this study was to gain a more complete understanding of the natural history of ERG changes in a common laboratory mouse strain. An important advantage of the present approach over most prior reports is that the same cohort of mice was followed in a longitudinal design, which helped to minimize inter-mouse differences as a source of variability. Additionally, ERGs were performed on a relative fine timescale to provide better insight into the time-course of ERG changes. The four primary findings of this study are (1) dark-adapted flash a- and b-wave amplitudes decreased similarly with age for all stimulus flash luminances examined; (2) light-adapted flicker ERGs decreased with age similarly for all stimulus temporal frequencies examined; 3) the response timing was less affected by age, as compared to the amplitude of the responses; 4) ocular impedance did not differ significantly for mice that were 8 and 70 weeks of age.

The decrease in dark-adapted flash a- and b-wave amplitudes (approximately a factor of 2) from 5 to 70 weeks of age was similar to that reported in previous studies. That is, Li et al.^8^ reported that the dark-adapted a- and b-wave amplitudes at 12 months were reduced by a factor of approximately 1.4, compared to the amplitude at 2 months of age. Of note, Li et al.^8^ found the amplitude differences due to age to be intensity-dependent, with the largest differences in age observed for the highest luminance flashes^8^. Likewise, Ferdous et al.^19^ reported statistically significant age-related amplitude loss only for high luminance flashes. By contrast, the dark-adapted flash responses shown in Figs. 1 and 2 suggest approximately similar amplitude losses for all flash luminance levels (on a logarithmic scale). Kolesnikov et al.^5^ reported that the a- and b-wave amplitudes at 2.5 years of age were reduced by a factor of approximately 1.5, compared to 4-month-old mice, and Gresh et al.^7^ found dark-adapted rod a- and b-waves amplitudes of C57BL/6 and BALB/c mice decreased by a factor of approximately two from 1 to 17 months of age.

The present study examined light-adapted flicker responses across a broad range of stimulus temporal frequency to evaluate age-related changes in cone-pathway function. The decrease in light-adapted fundamental flicker amplitude (a factor of 1.8) was similar to that reported by Williams and Jacobs^6^ who observed a loss of 12.5 Hz flicker ERG amplitude (a factor of approximately 2) over the age range of approximately 1 to 30 months in C57BL/6 mice. Gresh et al.^7^ reported a 1.6 × loss of photopic flicker amplitude for older mice (17 months), compared to younger mice (1 month). Their data suggested that lower temporal frequencies may be more affected by age than higher temporal frequencies, but frequency-dependent effects of age were not analyzed. For the first time, we report the effects of aging on the second harmonic amplitude of the mouse flicker ERG (a factor of 1.5 decrease over 5 to 70 weeks of age). The results of the present study show that age has highly similar effects on the fundamental and second harmonic ERG components across temporal frequency. Likewise, the changes in light-adapted flicker ERG amplitude with age (both the fundamental and second harmonic) were remarkably similar to those observed for the dark-adapted flash responses.

The explanation for the loss of ERG amplitude with age remains uncertain. The age-related decline of dark-adapted b-wave amplitude presented in Fig. 2, and in the supplemental material, could be due to number of factors including loss of photoreceptors. However, age-related photoreceptor loss in mice remains somewhat controversial. Li et al.^8^ reported no loss of receptor density from 2 to 12 months of age in C57BL/6 mice, and Trachimowicz et al.^20^ found similar rod densities in mice ranging in age from 2 to 32 months. Likewise, Williams and Jacobs^6^ reported no age-related change in opsin gene transcription or cone densities over the age range of 1–25 months. Gresh et al.^7^ and Ferdous et al.^19^, however, reported age-related decreases in outer nuclear layer (ONL) thickness. In aged B6D2F1/J mice (2.5 years of age), Kolesnikov et al.^5^ found that the number of rods and their outer segment lengths were significantly reduced compared with 4-month-old animals. They also observed an age-related reduction in the total level of opsin in the retina. Overall, it is challenging to reconcile these studies due to differences in mouse strain, ages examined, and particular structural assay performed. Further, the extent of age-related cone loss may depend on the area of the retina examined, as previous work^21^ has indicated significant M-cone loss only in the peripheral ventral retina in C57BL/6 mice.

The age-related decline of dark-adapted b-wave amplitude could also be due to disruption of the dark current, post-receptor dysfunction, or response compression^22^. Of note, local random dropout of rods would likely reduce rod b-wave sensitivity (elevate log *K*) with less effect on the maximum amplitude^3,22^, which was not observed (Supplemental Materials). Cataract and other pre-retinal media changes would also be expected to reduce sensitivity (elevate *K*). However, log *K* was found to be largely independent of age (Supplemental Materials), which suggests that optical factors are not likely responsible for the age-related b-wave amplitude loss. An age-related loss of log *V*_*max*_ with minimal effects on log *K* was also reported for dark-adapted human ERG b-waves^3^ and for mouse photopic flicker responses^6^.

It was hypothesized that a change in ocular impedance may account for the age-related ERG amplitude losses^8^. The ERG is a field potential, so a decrease in ocular impedance with age would be expected to decrease the ERG voltage recorded at the cornea, consistent with Ohm’s law. This proposal would parsimoniously account for the similar loss of rod- and cone-pathway-mediated ERG amplitudes. However, the results of the present study showed that ocular impedance was similar in mice that were 8 and 70 weeks of age, despite differences in rod- and cone-pathway ERG amplitude at these ages. Of note, we did not determine ocular impedance at other ages and it is possible that an increase in impedance may contribute to amplitude loss at older ages. We are not aware of previous reports of ocular impedance in mice for comparison, but Rahmani et al.^15^ reported that ocular impedance was approximately 40–50 kΩ in rats, consistent with the values shown in Fig. 6. In short, a decrease in ocular impedance with age does not appear to explain the age-related ERG amplitude loss, but future comprehensive analyses of impedance in mice are warranted.

The results of the present study may be of use in guiding future longitudinal pre-clinical therapeutic studies. Based on the present data set, the flicker and flash ERG amplitudes would not be expected to decline by more than a factor of approximately 2 over the course of 5 to 70 weeks. Amplitudes declines of more than a factor of 2 over this time range would suggest pathological loss. For shorter follow-up durations, amplitude losses smaller than a factor of two would be anticipated, and the data provided in the present report may assist in estimating the extent of normal amplitude loss across different ages. Age had a relatively small effect on the timing of the single flash responses and no significant effect on the flicker response phase. Thus, substantial changes in response timing over the course of 5 to 70 weeks would also suggest pathological processes. Importantly, the present study only included male mice from 5 to 70 weeks of age. Additional work is needed to determine if a similar effect of aging is observed in female mice and in older mice of either sex.

In summary, dark-adapted flash and light-adapted flicker ERG amplitudes similarly decreased in C57BL/6J mice as they aged from 5 to 70 weeks. At present, the explanation for the ERG amplitude loss over time remains uncertain. Future studies of retinal pigment epithelium (RPE) function, as well as ex-vivo ERG recordings, may provide useful insight into the source of age-related changes in the ERG. Regardless of the source of the age-related ERG amplitude loss, age should be considered an essential biological variable in ERG studies of C57BL/6 mice.

## Supplementary Information

Supplementary Figure S1.

## Data Availability

All data described in the current study are available from the corresponding author upon request.
