# Supplementary material for: Effects of normal aging on the mouse retina assessed by full-field flash and flicker electroretinography
Source: Sci Rep. 2023 May 31;13:8860. doi: 10.1038/s41598-023-35996-7 (PMC10232421; doi:10.1038/s41598-023-35996-7)
Supplement: Supplementary file 1 — Supplementary Figure S1. [file 41598_2023_35996_MOESM1_ESM.docx]

Supplemental Material; Figure S1: b-wave amplitude is plotted as a function of log stimulus luminance. Each symbol represents mean data obtained at a different age (replotted from Fig. 2), with the youngest mice (5-weeks) indicated by the black circles and the oldest mice (70-weeks) indicated by the green hexagons. The data are fit with Naka-Ruston functions: *V = V_max_ (L^n^/(L^n^+K^n^))*, where *V_max_* is the asymptotic b-wave amplitude, *L* is the stimulus luminance, *K* is the stimulus luminance necessary to elicit a half-maximum b-wave amplitude, and *n* is the slope (set to 1.0). Note that only luminance levels less than 0 log cd-s-m^-2^ that comprised the first limb of the response function were included in the fit. The second limb, which likely represents interference between the processes that generate the a- and b-waves, was excluded, per convention. It was assumed that the amplitude functions would extrapolate to approximately 0 µV at -4.0 log cd-s-m^-2^, an assumption that will affect log *K*.

The middle panel shows log *V_max_* as a function of age. Consistent with Fig. 2, *V_max_* decreased with age over approximately 5 – 30 weeks, slightly increased from weeks 30 – 50, then decreased sharply after 50 weeks of age.

The lower panel shows log *K* as a function of age. Log *K* was generally independent of age, differing by 0.06 log units at 5 and 70 weeks of age.
